# Improvement in Color-Conversion Efficiency and Stability for Quantum-Dot-Based Light-Emitting Diodes Using a Blue Anti-Transmission Film

**DOI:** 10.3390/nano8070508

**Published:** 2018-07-09

**Authors:** Jiasheng Li, Yong Tang, Zongtao Li, Xinrui Ding, Shudong Yu, Binhai Yu

**Affiliations:** 1 Engineering Research Center of Green Manufacturing for Energy-Saving and New-Energy Technology, South China University of Technology, Guangzhou 510640, China; jiasli@foxmail.com (J.L.); ytang@scut.edu.cn (Y.T.); ding.xinrui@mail.scut.edu.cn (X.D.); yu.shudong@mail.scut.edu.cn (S.Y.); mebhaiyu@scut.edu.cn (B.Y.); 2 Foshan Nationstar Optoelectronics Company Ltd., Foshan 528000, China

**Keywords:** light-emitting diodes, quantum dots, stability, color-conversion efficiency, photoluminescence

## Abstract

In this report, a blue anti-transmission film (BATF) has been introduced to improve the color-conversion efficiency (CCE) and the stability of quantum dot (QD) films. The results indicate that the CCE can be increased by as much as 93% using 15 layers of BATFs under the same QD concentration. Therefore, the same CCE can be achieved using BATF-QD hybrid films with a lower QD concentration when compared with standard QD films. The hybrid and QD films with the same CCE of 60% were aged at an environmental temperature of 25°C and with a 10 mA injection current light-emitting diode source. The CCE and luminous efficacy that are gained by the hybrid film increased by 42.8% and 24.5%, respectively, when compared with that gained by the QD film after aging for the same time period of approximately 65 h. In addition, the hybrid film can effectively suppress the red-shift phenomenon of the QD light spectra, as well as an expansion of the full-width at half maximum. Consequently, these BATF-QD hybrid films with excellent optical performance and stability show great potential for illumination and display applications.

## 1. Introduction

Quantum dots (QDs) have several advantages for lighting applications, including high quantum yield, high color purity, and easy manufacture. They have demonstrated great potential in optoelectronic devices, such as light-emitting diodes [1]. Blue LED chips are generally utilized to excite QD films. In this process, the mixing of the blue light from the LEDs (chip light) and the conversion light from the QDs (QD light) can achieve different output colors [2]. Significant effort has been directed at attempting to improve the quantum yield (QY) of QDs by optimizing the core/shell structures [3] and the surface functional groups [4]. At present, the QY of CdSe/ZnS is already in excess of 90% [5]. This OD is widely regarded as one of the most promising candidates for replacing traditional rare-earth-based phosphor materials [6]. However, significant challenges are still encountered when these QDs are used in LEDs. Similar to other phosphor materials, such as yttrium aluminitum garnet (YAG) and nitride phosphor, it is necessary to disperse the QDs into a transparent matrix in order to achieve high light extraction [7] and to simultaneously prevent oxidation [8]. QDs are easy to aggregate in a matrix due to their small particle size of several nanometers. However, this results in aggregation induced quenching (AIQ) [9], which leads to high conversion losses. Some polymer matrixes, including polyvinyl alcohol (PVA) [10], polydimethylsiloxane (PDMS) [11], and gel glass [12] have been proposed to improve the dispersity of QDs, thereby decreasing this loss. In addition, QDs strongly absorb their emission light, which results in significant reabsorption loss [13,14,15]. This may lead to larger thermal power generation from the QDs and a reduced working stability [16].

The color-conversion efficiency (CCE) refers to the proportion of QD light radiant power to the total radiant power. It determines the output color coordinates of LEDs [17,18]. This is one of the most essential parameters when characterizing illuminations and displays. It is common to increase the CCE by increasing the QD concentration in the QD matrix [19]. However, a larger QD concentration can significantly increase AIQ and the reabsorption by the QDs [15], leading to a lower conversion efficiency of the QD films when compared to the QY of the QDs. Therefore, the luminous efficacy of the QD-based LEDs is still significantly less than that of traditional phosphor-based LEDs, especially for high CCE LEDs [20]. To solve this issue, the scattering effect that is introduced by particles with a high refractive index [21,22] or patterned structures [18] is used to improve the CCE by increasing the blue light path in the QD films. However, these methods also increase the light path of the QD light in the QD films, leading to higher reabsorption loss and heat power generation from the QDs [23]. Consequently, it is expected that any new solution for improving the CCE should simultaneously decrease reabsorption losses [20]. Solving this problem is an important aspect in the development of high efficiency and stable QD-based LEDs.

In this study, a blue anti-transmission film (BATF) is introduced in order to improve the CCE and stability of QD films. The absorption of QD films and BATF-QD hybrid films were investigated. Then, the influence of the BATF-QD hybrid films on the optical performance of LEDs was investigated. Finally, the optical performance and working stability of the BATF-QD hybrid films were compared with QD films under the same CCE.

## 2. Methods 

**Figure 1:**
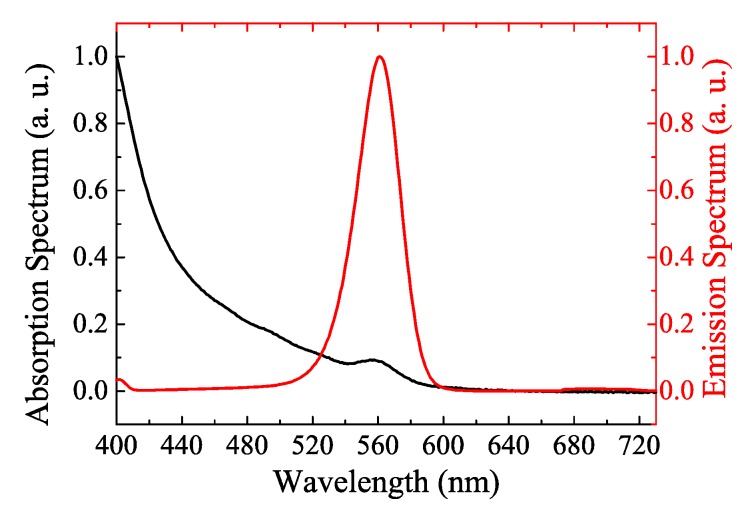
Absorption and emission spectra of CdSe/ZnS quantum dots (QDs) solution.

**Figure 2:**
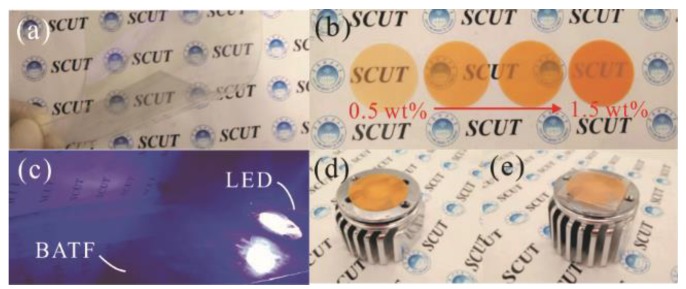
The (**a**) BATF; (**b**) QD films; (**c**) blue light spot reflection by BATFs; (**d**) LED assembled with a QD films; and (**e**) LED assembled with a BATF-QD hybrid film (5 layers of BTAFs).

The QDs with the CdSe/ZnS core/shell structure (purchased from China Beijing Beida Jubang Science & Technology Co., Ltd., Beijing, China, quantum yield larger than 80%) were used in this study. The emission and absorption properties of these QDs in chloroform solution are shown in Figure 1. PDMS (Sylgard-184, purchased from Dow Corning, Midland, MI, USA) was used as the transparent matrix to disperse the QDs. Since the blue light is necessary for the illumination and display applications, we have selected the BATF with a specific blue light transmittance instead of entirely absorbing the blue light. The commercial BATFs with approximately 70% blue light transmittance are widely used for eye-protection in screens of mobile phones and computers, which have been used in this study. These BATFs were purchased from Shen Zhen Fancy Package & Manufactory Co., Ltd. (Shenzhen, China). The blue blocking function is realized by the multilayer composites with gradient refractive index (the total thickness of the functional composites is 100 μm). The transparent substrate is PET polymer, as shown in Figure 2a. They strongly reflect blue light, as shown in Figure 2c, and are widely used as eye-protection films in screens. The LED source has a radius of 8 mm, and was packaged as 42 blue LED chips with an emission wavelength centered at 455 nm; all of the LED chips were sealed by the silicone-based encapsulant; the electrical injection power is 150 mW (injection current of 10 mA), which is a harsh condition for QD-based LED operation [24]. The QD films were fabricated using the evaporation-curing method [11,22,23]. Firstly, QDs were added into 2 mL of chloroform solution to uniformly disperse them in the solution systems with low viscosity; the total mass of the QDs were 10 mg, 15 mg, 20 mg, and 30 mg, in order to control the QD concentration in the QD films. After mixing for several seconds, 909 mg of pre-polymer A-PDMS was added to the QD-chloroform solution. This mixture was stirred for 40 mins until the chloroform solution completely evaporated. Then, 91 mg of curing agent of B-PDMS was added into the mixtures, which were stirred and evacuated for 15 mins. Finally, the QD-PDMS mixtures were injected into a mold and then cured at a temperature of 120 °C for 90 mins. QD films with a thickness of 0.5 mm and radius of 16 mm were produced. The BATF-QD hybrid films can be easily fabricated by placing the BATFs inside the mold during the curing process. Moreover, the BATFs were stamped to each other using PDMS in order to achieve multi-layer BATFs. The optical properties of the BATFs, QD films, and BATF-QD hybrid films were measured using a dual-beam UV-Vis spectrophotometer TU-1901 (Beijing Persee General Instrument Co., Ltd., Beijing, China). These films were also assembled on an LED source, and then their optical performance were measured using the Integrating Systems from Instrument Systems (Munich, Germany). The injection current of 10 mA is provided by a source from Keithley (Beaverton, OR, USA). During measurements, it should be noted that the BATFs are always on the upper side of the QD film.

The working stability was investigated according to the aging tests at an environmental temperature of 25 °C without heat sink for thermal managements [24]. The BATF-QD hybrid films and QD films were assembled to the same LED source mentioned above, respectively. The LED source was kept with an injection current of 10 mA (electrical injection power of 150 mW) to continuously excite these QDs films for degradations. The optical performance of LEDs assembled with BATF-QD hybrid films and QD films were measured at different aging time using the same injection current of 10 mA.

## 3. Results and Discussion

**Figure 3:**
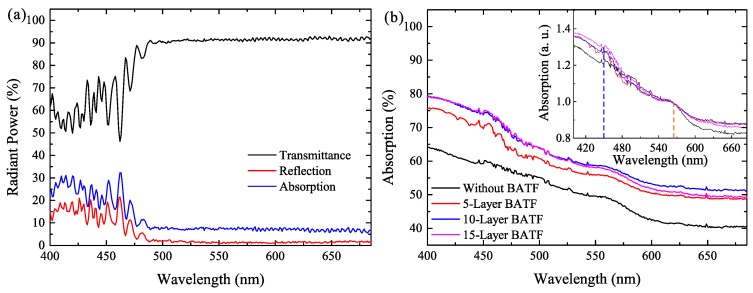
(**a**) The transmittance, reflection, and absorption spectrum of the BATF; and (**b**) the absorption spectra of the QD films and the BATF-QD hybrid films.

The optical properties including wavelength-dependent transmittance, reflection, and absorption of the commercial BATF were characterized, as shown in Figure 3a. The BATF has a low average transmittance of 69.1% for blue light with a wavelength less than 500 nm, which is due, in part, to its high reflection (average of 11.3%) and absorption (average 19.6%) for these wavelengths. Moreover, it exhibits almost no reflection for wavelengths that are longer than 500 nm. The average transmittance is as high as 91.5%, while the absorption is approximately 7% for these wavelengths. These values indicate that BATF is beneficial with respect to suppressing the transmittance of chip light (emission from LEDs), while also introducing some additional absorption loss for QD light (emission from QDs). Figure 3b shows the absorption spectra of the QD films and the BATF-QD hybrid films with different layers of BATFs. The concentration of the QD is kept as 0.75 wt. %. The BATF-QD hybrid films have a higher absorption when compared with the QD films, and the absorption becomes larger with additional layers of BATFs. Besides, it shows less increment in the absorption as the number of BATF layers increases. This is because much more reflection light can escapes from the lateral side of BATFs with a larger thickness instead of being absorbed by the QD film. The inset figures of Figure 3b show the absorption spectra when normalized to the absorption at the peak emission wavelength of the QDs. It can be observed that the BATF-QD hybrid films have a higher absorption for blue light (at 455 nm) when they have the same absorption for the peak wavelength of the QD light, especially for the hybrid films with several layers of BATFs. This implies that the BATF-QD hybrid films are useful in reducing the chip light with increasing layers of BATFs, while maintaining a large amount of QD light, which may be beneficial to increasing the CCE of the QDs.

**Figure 4:**
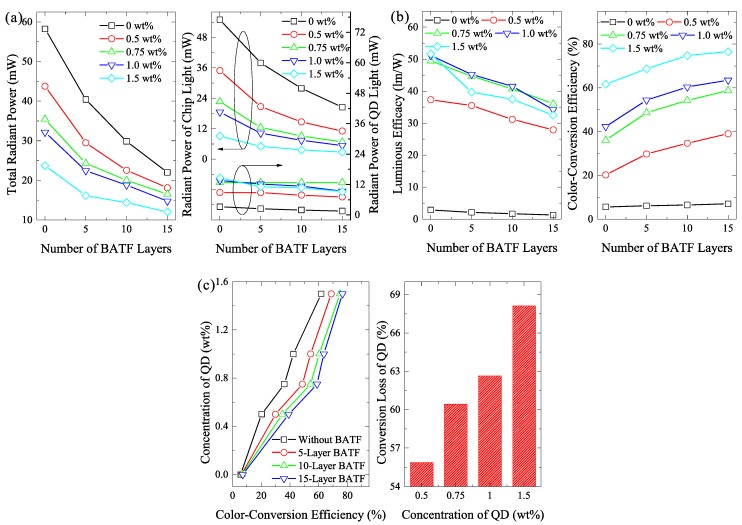
The (**a**) total radiant power, radiant power of the chip light, radiant power of the QD light; (**b**) luminous efficacy, and color-conversion efficiency of LEDs with different QD concentrations and BATF layers; (**c**) the QD concentration verse color-conversion efficiency with different BATF layers and the conversion loss with different QD concentration. The QD concentration is various from 0 wt. % to 1.5 wt. %.

The QD film and BATF-QD hybrid film with different layers of BATFs and different concentrations of QDs were applied to a blue LED source. The radiant power, including the total radiant power, radiant power of chip light, and radiant power of QD light, of these QD-based LEDs is given in Figure 4a. It should be noted that the radiant power of the chip light and the QD light were obtained by integrating the emission radiant spectra of QD-based LEDs from 380 nm to 525 nm and from 525 nm to 730 nm, respectively. As the number of BATF layers was increased, the total radiant power significantly decreases. This demonstrates that the use of BATF can lead to a reduction in light extraction due to its high reflection and absorption. However, this decrease becomes smaller as the QD concentration increases. When the number of BATF layers increases from 0 to 15, the total radiant power that is gained for a QD concentration of 0 wt. % decreases by 62.2%. There is a decrease of 49.2% for a QD concentration of 1.5 wt. %. One reasonable explanation for this observation is that much more chip light is absorbed by the QD films prior to propagating into the BATFs as the QD concentration is increased. This results in more QD light with a higher transmittance. These results are also supported by the significant reduction in the radiant power of the chip light as the QD concentration increases. Although the BATF has a high absorption for QD light that is comparable with that of chip light, it is interesting that the radiant power shows a significant decrease with increasing BATF layers. Only a slight change is observed for the radiant power of the QD light. These results indicate that a portion of the chip light reflected by BATF has propagated into the QD films and it is absorbed by QDs, thereby increasing the excitation of the QD light. In other words, it is the reuse of the reflected chip light that prevents the reduction in the radiant power of the QD light.

Their luminous efficacy and CCE are given in Figure 4b. Similarly, the luminous efficacy also decreases with increasing BATF layers due to the absorption and reflection losses that are introduced by the BATFs. However, the reduction in the luminous efficacy is smaller than 37%, which is less than that of the radiant power. This is because the luminous efficacy is more sensitive to the QD light, which still maintains a high radiant power. Most importantly, the CCE shows a significant increase as the number of BATF layer increases. This increase is more significant at lower QD concentrations. An increase of 93% using 15 layers of BATFs is observed when the QD concentration is 0.5 wt. %. This is because when the QD film has a lower concentration, it more readily facilitates the escape of QD light due to the lower probability of reabsorption events.

Moreover, it is interesting that LEDs using BATF-QD hybrid films with a lower QD concentration can attain the same CCE as QD films with larger QD concentrations. To further investigate this issue, the results for the QD concentration for the same CCE when a different number of BATF layers were used, are given in Figure 4c. It is evident that more BATF layers are beneficial to decreasing the QD concentration at the same CCE. For example, when 15 layers of BATFs are used, a QD concentration of only 0.75 wt. % can result in a CCE that is as high as that of 1.5 wt. % without using BATFs. The conversion loss of the QDs is also given in Figure 4c, which was calculated using the equation (PCabs−PQDemit)/PCabs. PCabs and PQDemit are the QD absorption power of the chip light and the radiant power of the QD light, respectively. It is obvious that a larger QD concentration can cause higher conversion losses due to significantly more reabsorption events. The conversion loss gained with a 0.75 wt. % QD concentration can decrease by 11.3% when compared with that gained using 1.5 wt. % QD concentration. Therefore, the BATF-QD hybrid films can achieve a high CCE using a low QD concentration, which is beneficial to decreasing conversion losses inside QD films.

**Figure 5:**
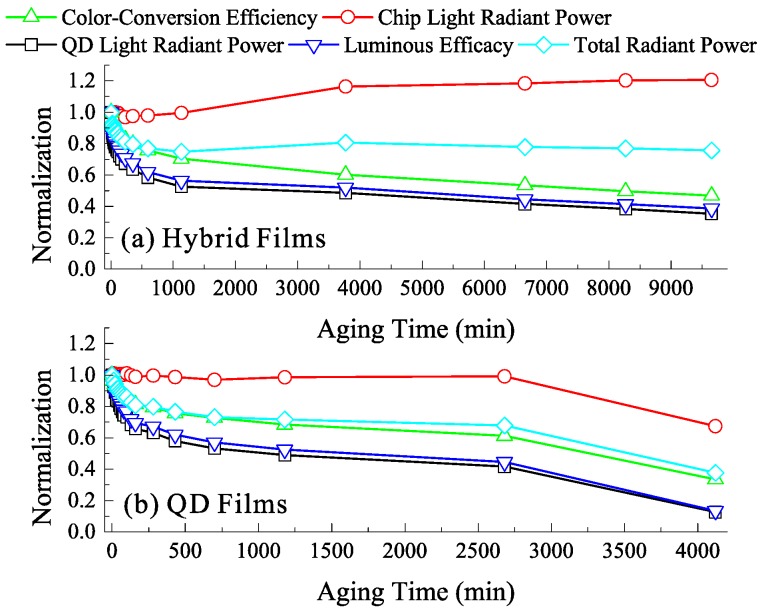
The optical performances of LEDs using (**a**) hybrid films (0.75 wt. % QD concentration and 15-layer BATFs) and (**b**) QD films (1.5 wt. % QD concentration) with different aging time, respectively.

**Figure 6:**
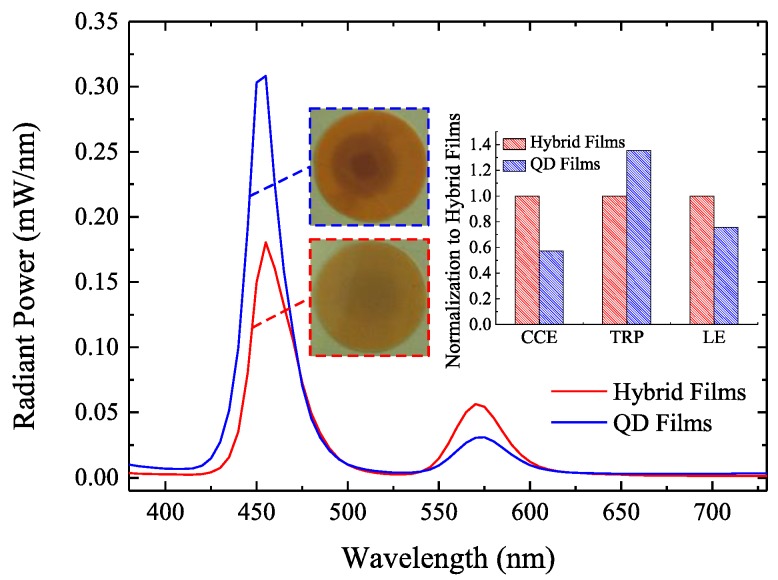
The emission spectra of LEDs using hybrid films (0.75 wt. % QD concentration and 15-layer BATFs) and QD films (1.5 wt. % QD concentration) after aging for approximately 65 h. The inset figure represents their color-conversion efficiency (CCE), total radiant power (TRP), and luminous efficacy (LE) normalized to that of the BATF.

The aging test was performed to evaluate this issue, the optical performances of LEDs using hybrid films (QD concentration of 0.75 wt. %, 15-layer BATFs, initial CCE of 60%) and QD films (QD concentration of 1.5 wt. %, initial CCE of 60%) with different aging time are shown in Figure 5a,b, respectively. The optical performances including the radiant power, luminous flux, and CCE of these LEDs dramatically decrease at the initial stage (within 200 min), which is similar to previous investigations on their stability [16,24]. One possible explanation is that much more QD light at the initial stage leads to much more reabsorption events between QDs, resulting in greater thermal power generation from QDs [20,25]. However, it is evident that the luminous efficacy that is gained by the QD film significantly decreases when compared with the BATF-QD hybrid film, although they have the same initial CCE of 60%. As discussed above, this is because that the BATF significantly reduces the chip light, while maintaining a large amount of QD light, which realizes the same CCE for LEDs using the hybrid film with a lower QD concentration compared with that only using the QD film with a higher QD concentration. A lower QD concentration leads to less reabsorption loss [20,26], thereby decreasing the thermal power generating from QDs and improving the stability. Their stability have been compared in detail. After aging for approximately 65 h, the CCE, total radiant power, and luminous efficacy gained by the QD film decrease by 66.5%, 62.4%, and 86.6%, respectively; while, that gained by the hybrid film only decreased by 39.7%, 19.4%, and 48%, respectively. Their spectra after aging for approximately 65 h are also given in Figure 6. It is interesting that the radiant power of the spectra of the QD light that are acquired from the hybrid film are even higher than that acquired from the QD film. As such, the hybrid film can have a higher CCE and luminous efficacy when compared to the QD film after aging. These values are increased by 42.8% and 24.5%, respectively, as shown in the inset figure. It should be noted that the initial luminous efficacy (before aging) of this hybrid film is 30% lower than that of the QD film at the same CCE of 60%, as shown in Figure 4. However, this property is significantly increased by 24.5% after aging. This indicates that the hybrid film has a lower conversion loss and generates less thermal power, which avoids the destruction of the QDs and silicone carbonization [25]. Therefore, the BATF-QD hybrid film appears to result in a better working stability and better optical performances after aging. In addition, the initial luminous efficacy using these hybrid films is far lower than that using the QD films at the same CCE. This is mainly because the absorption losses that are introduced by the BATFs are much larger than the reduction in the conversion loss. Consequently, it is necessary to modify the BATF structures in order to simultaneously improve light extraction of the LEDs. This will help to further improve the optical performances of these hybrid films. To avoid misinterpretation, it should be noticed that the percentage improvements given above are arbitrary and not absolute.

**Figure 7:**
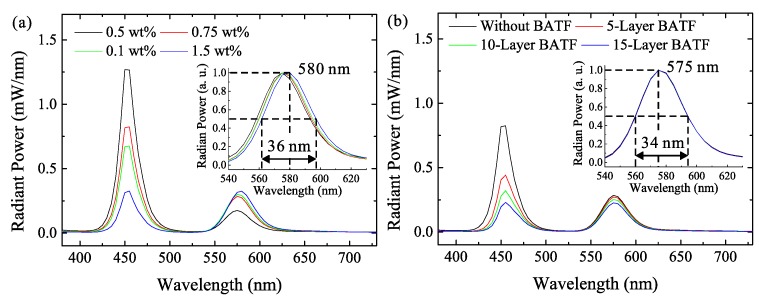
The emission spectra of LEDs using (**a**) QD films with different QD concentration and (**b**) hybrid films with 0.75 wt. % QD concentration and different BATFs layers.

The spectra of LEDs using QD films and BATF-QD hybrid films with different QD concentrations and BATF layers are further investigated, as given in Figure 7. Figure 7a shows the spectra that are acquired from the QD films. The radiant power of the spectra of the QD light increase with an increasing QD concentration, while it should be noted that their increment is significantly low when compared with the reduction in the radiant power of the spectra of the chip light, especially when the QD concentration is sufficiently large. This is because of the severe reabsorption losses of the QD films with high QD concentration, as previously indicated. Besides, the reabsorption effect leads to a red-shift of the spectra, as shown in the normalized spectra in the inset figures. When the QD concentration is 1.5 wt. %, the peak wavelength in the spectra of QD light shifts to 580 nm, and its full-width at half maximum (FWHM) is increased to 36 nm. As a result, although the increase in the QD concentration can result in an increase of the CCE, it also causes the spectra to be unpredictable. The spectra that are acquired from the BATF-QD hybrid films for a QD concentration of 0.75 wt. % are shown in Figure 7b. The spectra of the chip light is significantly decreased, while that of the QD light shows a slight decrease with an increasing number of BATF layers. This is beneficial to increasing the CCE, as previously discussed. However, it is interesting that their normalized spectra show almost no change. It is noteworthy that the CCE gained using 0.75 wt. % QD concentration and 15 layers of BATFs is almost the same as that gained by QD films using 1.5 wt. % QD concentration. The peak wavelength and the FWHM of the QD light are 575 nm and 34 nm, respectively. These values are the same as the values that are acquired from the QD films using 0.75 wt. % QD concentration and smaller than the values acquired from the QD films for 1.5 wt. % QD concentration. Therefore, compared with QD films with a higher QD concentration under the same CCE, the BATF-QD hybrid films with a lower QD concentration are also beneficial for suppressing the red-shift phenomenon. This is helpful for accurately obtaining color coordinates, which is critically important in illuminations and display applications. 

## 4. Conclusions

In this report, BATF was introduced to improve the CCE and stability of QD films. The results show that the BATF-QD hybrid films significantly increase the CCE by 93% using 15 layers of BATFs with the same QD concentration. This is caused by the stronger absorption and reflection of chip light. The same CCE (as high as 60%) can be achieved using hybrid films with a QD concentration half that of QD films, although the initial luminous efficacy (before aging) acquired by the hybrid film was substantially lower than that acquired by the QD film with the same CCE of 60%. After aging for approximately 65 h, the CCE and luminous efficacy gained by the hybrid film increased by 42.8% and 24.5% when compared with that of the QD film. These results demonstrate that a hybrid film with a lower QD concentration can significantly decrease conversion losses due to the reduced probability of reabsorption events. As a result, the stability and optical performance are significantly increased after aging. Because of the lower QD concentration usage, the hybrid film can also effectively suppress the red-shift phenomenon of the spectra, which simultaneously leads to a smaller FWHM of the QD light. Therefore, the BATF-QD hybrid films show great potential in the illumination and display applications.

In future investigations, we intend to optimize the BATF structure to decrease its absorption while increasing its reflection. We believe that this will further improve the light extraction of LEDs by avoiding the absorption losses introduce by the BATFs.
